# Ethoxysanguinarine, a Novel Direct Activator of AMP-Activated Protein Kinase, Induces Autophagy and Exhibits Therapeutic Potential in Breast Cancer Cells

**DOI:** 10.3389/fphar.2019.01503

**Published:** 2020-01-08

**Authors:** Yuan Si, Jiu Wang, Xuewen Liu, Tong Zhou, Yuchen Xiang, Te Zhang, Xianhui Wang, Tingting Feng, Li Xu, Qingqing Yu, Huzi Zhao, Ying Liu

**Affiliations:** ^1^Laboratory of Molecular Target Therapy of Cancer, Institute of Basic Medical Sciences, Hubei University of Medicine, Shiyan, China; ^2^Laboratory of Molecular Target Therapy of Cancer, Biomedical Research Institute, Hubei University of Medicine, Shiyan, China; ^3^Hubei Key Laboratory of Wudang Local Chinese Medicine Research and Institute of Medicinal Chemistry, Hubei University of Medicine, Shiyan, China; ^4^Hubei Key Laboratory of Embryonic Stem Cell Research, Hubei University of Medicine, Shiyan, China

**Keywords:** ethoxysanguinarine, breast cancer, autophagy, AMP-activated protein kinase, molecular docking, pharmacokinetic

## Abstract

Ethoxysanguinarine (Eth) is a benzophenanthridine alkaloid extracted from *Macleaya cordata (Willd) R. Br*. It possesses antibacterial and antiviral activities and offers therapeutic benefits for the treatment of respiratory syndrome virus-induced cytopathic effects. However, the effect of Eth on human tumors and its pharmacological effects remain to be elucidated, together with its cellular target. Here, we examined the effects of Eth on breast cancer (BC) cells. We found that at low doses, Eth strongly inhibited the viability of BC cell lines and induced autophagy. Mechanistic studies showed that Eth induced autophagy by upregulating the activity of the AMP-activated protein kinase (AMPK). The AMPK inhibitor compound C significantly attenuated Eth-induced autophagy and inhibited proliferation. Meanwhile, the AMPK activator metformin significantly enhanced Eth-induced autophagy and inhibited proliferation. Computational docking and affinity assays showed that Eth directly interacted with the allosteric drug and metabolite site of AMPK to stabilize its activation. AMPK was less activated in tumor samples compared to normal breast tissues and was inversely associated with the prognosis of the patients. Moreover, Eth exhibited potent anti-BC activity in nude mice and favorable pharmacokinetics in rats. These characteristics render Eth as a promising candidate drug for further development and for designing new effective AMPK activators.

## Introduction

Breast cancer (BC) is the most frequently diagnosed cancer and the leading cause of cancer death among females worldwide, with an estimated 2,088,849 cases and 626,679 deaths in 2018 (Bray et al., 2018). In China, BC is the leading cause of cancer death in women younger than 45 years. Based on reports in 2015 from the cancer statistics in China, 70.7 persons per 100,000 die annually from BC (Chen et al., 2016). The major treatment methods for BC patients are surgery, radiotherapy, and chemotherapy. Despite recent advances in diagnosis and treatment, BC mortality rates are still high, making newer and more advanced therapies indispensable. The development of novel agents for the prevention and treatment of human BC is therefore highly desirable.

Autophagy is a catabolic process for the degradation and recycling of macromolecules and organelles, which is characterized by the sequestration of bulk cytoplasm and organelles in double- or multi-membrane autophagic vesicles and their subsequent degradation by lysosomes for macromolecular synthesis and adenosine triphosphate (ATP) generation (Liang et al., 2017). Many studies have demonstrated that autophagy is not only a survival response to either growth factors or nutrient deprivation but also an important molecular mechanism for tumor cell suicide (Shi et al., 2012; Zhang et al., 2014). The molecular mechanism underlying the induction of autophagy in cancer has not been completely described, but so far, more than 30 autophagy-related genes (ATGs) have been identified (Zhang et al., 2017). Upstream of the *ATG*s, mammalian target of rapamycin (mTOR) kinase has the most potent impact on autophagy (Ebrahim et al., 2018). mTOR complex 1 (mTORC1) promotes protein synthesis, lipid biogenesis, cell growth, and anabolism and inhibits cellular catabolism by preventing autophagy. Once mTORC1 is activated, it inhibits autophagy *via* phosphorylation of the ATG proteins (Wan and Liu, 2019). Many studies have shown that AMP-activated protein kinase (AMPK) activation leads to autophagy through the negative regulation of mTORC1 (Shi et al., 2012; Chen et al., 2017). AMPK is a heterotrimer enzyme composed of one catalytic subunit (α1 or α2), one scaffolding subunit (β1 or β2), and one regulatory subunit (γ1, γ2, or γ3). Full AMPK activation requires the specific phosphorylation of the α subunit at Thr172. AMPK is most widely known for its role as an energy state sensor. Upon activation, AMPK induces a series of metabolic changes to maintain the production of intracellular energy and balance consumption (Kurumbail and Calabrese, 2016). Recent studies have shown that AMPK is a possible autophagy-associated tumor suppressor for the prevention and treatment of several cancer types (Han et al., 2018; Zhang et al., 2018; De Veirman et al., 2019). Accordingly, AMPK activators have been discovered as potential targeted drugs for the treatment of human cancer, and there is a need to develop novel AMPK activators with a low toxicity and high efficiency for inducing tumor cell autophagic suicide.

**Figure 1:**
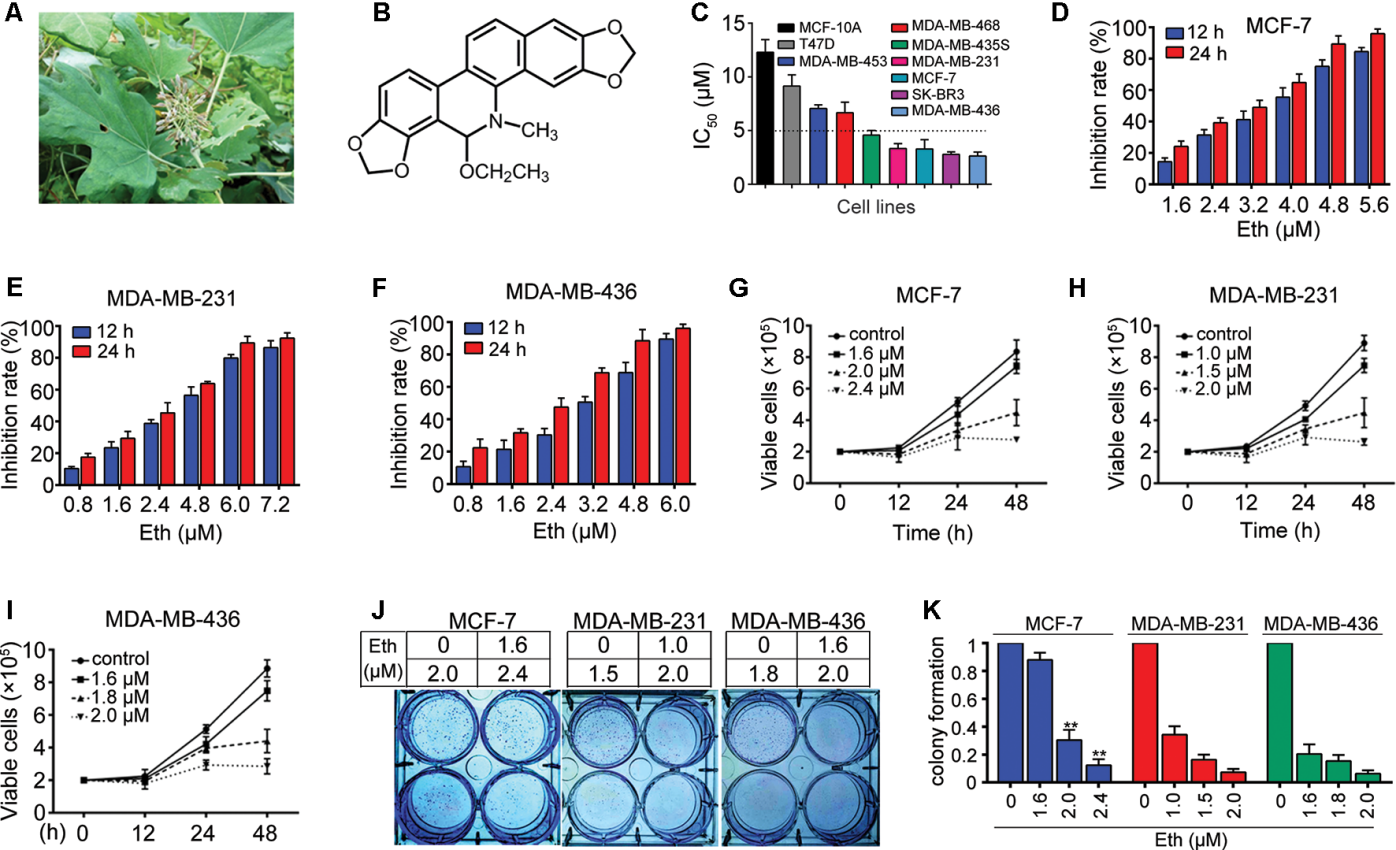
Eth inhibits BC cells. **(A)**: *M. cordata* image. **(B)**: Chemical structure of Eth. **(C)**: The IC_50_ of Eth for indicated cell lines. **(D**–**F)**: The inhibitory effects of Eth on MCF-7, MDA-MB-231, and MDA-MB-436 cells analyzed by MTT assay. **(G**–**I)**: Inhibitory effects of Eth on cell viability of MCF-7, MDA-MB-231, and MDA-MB-436 cells assayed by trypan blue exclusion assay. **(J**–**K)**: The colony formation assays of MCF-7, MDA-MB-231, and MDA-MB-436 cells treated with Eth at indicated concentration. **P < 0.01.

*Macleaya cordata (Willd.) R. Br*. (**Figure 1A**), also known as Bo Luo Hui in China, belongs to the *Papaveraceae* family (Huang et al., 2018). It is an herbaceous perennial plant that is ubiquitously dispersed in central China and has been used as traditional Chinese medicine for thousands of years. *M. cordata* has a variety of therapeutic uses for anti-fungal, anti-microbial, anti-inflammatory, anti-oxidant, and anti-tumor activities (Kosina et al., 2010; Ouyang et al., 2010; Yao et al., 2010; Cai et al., 2016). In Europe, North America, and China, *M. cordata* is also used to treat skin infections and insect bites (Cai et al., 2016). *M. cordata* is rich in various alkaloids, including sanguinarine, dihydroderivative, chelerythrine, protopine, allocryptopine, and phenolic acids (Ni et al., 2016; Lin et al., 2018). Ethoxysanguinarine (Eth, **Figure 1B**) is a product of the transformation of sanguinarine by crystallization of ammoniated ethanol during the extraction process (Konda et al., 1991). There are limited reports on the effect of Eth on cancer cells. In 2018, we revealed that Eth can induce inhibitory effects and downregulate the oncoprotein CIP2A (cancerous inhibitor of protein phosphatase 2A) in colorectal cancer cells (Jin et al., 2018). The effect and mechanism of Eth in other cancer types needs investigation. This study investigated the antitumor effects and possible mechanisms of Eth against BC.

## Materials and Methods

### Patients

Two independent BC cohorts tissue microarray (TMA) were utilized in this study. The training cohort TMA was purchased from Wuhan Iwill Biological Technology Co., Ltd. (Wuhan, China). It included 143 patients’ tissues and 36 paired non-cancerous normal tissues from these patients were obtained. The array dot diameter was 1.5 mm, and each dot represented a tissue spot from one individual specimen that was selected and pathologically confirmed.

### Immunohistochemistry of TMA

Immunohistochemical analysis as well as the scoring of immunoreactivity was performed using the rabbit monoclonal anti-pAMPKα (Thr172) antibody. The intensity of pAMPKα staining was scored as 0 (no signal), 1 (weak), 2 (moderate), and 3 (marked). Percentage scores were assigned as 1, 1–25%; 2, 26–50%; 3, 51–75%; and 4, 76–100%. The scores of each tumor sample were multiplied to give a final score of 0–12, and the tumors were finally determined as negative (−), score 0; lower expression (+), score ≤4; moderate expression (++), score 5–8; and high expression (+++), score ≥9. Tumor sample scored (+) to (+++) were considered positive (overexpression). An optimal cutoff value was identified: a staining index of 5 or greater was used to define of high expression and 4 or lower for low expression.

### Reagents

Eth with a purity of up to 98% was purchased from Shanghai Yuanye Bio-Technology Co., Ltd. (Shanghai, China). Eth was dissolved in DMSO (Sigma) at a stock solution of 50 mM and stored at –20°C. Biotinylated Eth (purity > 95%) was synthesized by Boshixing Synthetic Technologies, Inc. (Shenzhen, China).

### Cell Culture

Human BC cell lines MCF-7, MDA-MB-231, DMA-MB-436, SK-BR3, MDA-MB-468, MDA-MB-453, and MDA-MB-435S and non-tumorigenic MCF-10A human mammary epithelial cells were obtained from American Type Culture Collection (ATCC; Manassas, VA, USA). MCF-7, SK-BR3, MDA-MB-231, and MDA-MB-436 cells were maintained in Dulbecco’s modified Eagle’s medium (DMEM; high glucose; Gibco; Thermo Fisher Scientific, Inc., Waltham, MA, USA) supplemented with 10% fetal bovine serum (FBS; HyClone; Logan, UT, USA) and antibiotics and incubated in a humidified atmosphere with 5% CO_2_ at 37°C. MDA-MB-468, MDA-MB-453, and MDA-MB-435S cells were maintained in Leibovitz’s L-15 (Gibco) supplemented with 10% FBS and antibiotics and incubated in a humidified atmosphere without CO_2_ at 37°C. MCF-10A cells were maintained in DMEM/F12 medium containing 5% horse serum (HS), insulin (10 mg/mL), epidermal growth factor (EGF, 20 ng/mL), choleratoxin (100 mg/mL), hydrocortisone (0.5 mg/mL), penicillin (50 U/mL), and streptomycin (50 U/mL), and incubated in a humidified atmosphere with CO_2_ at 37°C.

### Cytotoxic Assay and Cell Viability

Cells were seeded into 96-well plate and pre-cultured for 24 h, then treated with Eth for 24. Cell cytotoxicity was determined by MTT assay. The absorbance was measured at 490 nm by automated microplated reader (BioTek Instruments, Inc., Winooski, VT, USA), and the cell death rate was calculated as followed: inhibition rate (%) = (average A_490_ of the control group − average A_490_ of the experimental group)/(average A_490_ of the control group − average A_490_ of the blank group) × 100%. Cell viability was estimated by trypan blue dye exclusion.

### Soft-Agar Colony Formation Assay

Cells were suspended in 1 mL of L-15 or DMEM containing 0.3% low-melting-point agarose (Amresco, Cleveland, Oh, USA) and 10% FBS, and plated on a bottom layer containing 0.6% agarose and 10% FBS in 6-well plate in triplicate. After 2 weeks, plates were stained with 0.2% gentian violet and the colonies were counted under light microscope (IX70; Olympus Corporation, Tokyo, Japan) after 2 weeks.

### Autophagy Assays

The cells were transfected with pQCXIP-GFP-LC3 plasmid using the Lipofectamine 3000 (Invitrogen; Thermo Fisher Scientific, Inc.) according to the recommended protocol by the manufacturer and then fixed in 4% paraformaldehyde. The percentage of cells with fluorescent dots representing GFP-LC3 translocation was counted. For visualization of cell nucleus, DAPI was used. Sections were observed using an Olympus laser scanning confocal microscope with imaging software (Olympus Fluoview FV-1000, Tokyo, Japan).

### Western Blot

Cells were lysed in radioimmunoprecipitation assay buffer containing 50 mM Tris, pH 8.0, 150 mM NaCl, 0.1% SDS, 0.5% sodium deoxycholate, 1% NP-40, 1 mM DTT, 1 mM NaF, 1 mM sodium orthovanadate, 1 mM phenylmethylsulfonyl fluoride (PMSF; Sigma-Aldrich; Merck KGaA), and 1% protease inhibitors cocktail (EMD Millipore, Billerica, MA, USA). Protein concentration was determined using the Bradford method. Equal amounts of sample (25 µg) were separated by SDS-PAGE (8–12% gels). Electrophoresed proteins were then transferred onto polyvinylidene fluoride membranes (EMD Millipore, Billerica, MA, USA). The membranes were blocked with 5% skimmed milk in Tris-buffered saline at room temperature for 1 h. Following blocking, membranes were incubated overnight at 4°C with primary antibodies and then rinsed with Tris-buffered saline with Tween 20. The following primary antibodies were used: anti-Glut3 (1:500; catalog no. sc-74399) (Santa Cruz Biotechnology, Inc., Dallas, TX, USA); anti-LC3 (1:1000; catalog no. 12741), anti-AMPKα (1:1000; catalog no. 2532), anti-phospho-AMPKα (Thr172) (1:1000; catalog no. 2535), anti-phospho-AMPKβ1 (Ser108) (1:1000; catalog no. 4181), anti-AMPKγ1 (1:1000; catalog no. 4187), anti-mTOR (1:1000; catalog no. 2983), anti-phospho-mTOR (Ser2448) (1:1000; catalog no. 5536), anti-P70S6K (1:1000; catalog no. 9202), anti-phospho-P70S6K (Thr389) (1:1000; catalog no. 92775), anti-4E-BP1 (1:1000; catalog no. 9234), anti-phospho-4E-BP1 (Thr37/46) (1:1000; catalog no. 2855), anti-ACC (1:1000; catalog no. 3676), anti-phospho-ACC (Thr79) (1:1000; catalog no. 11818), anti-Glut1 (1:1000; catalog no. 12939), anti-Glut4 (1:1000; catalog no. 2213) (All Cell Signaling Technology, Inc., Danvers, MA, USA), and anti-GAPDH (1:5000; catalog no. M20006; Abmart, Shanghai, China). Membranes were then washed, and incubated with horseradish peroxidase-conjugated secondary antibody (1:10000; E030120-01 and E030110-01; EarthOx, LLC, San Francisco, CA, USA) for 1.5 h at room temperature. The protein bands were visualized using SuperSignal^®^ West Pico PLUS Chemiluminescent substrate (catalog no. 34579; Pierce; Thermo Fisher Scientific, Inc., Rockford, IL, USA) (Zhang et al., 2018).

### AMPK Activity Assay

AMPK kinase activity was assay was performed by AMPK kinase activity kit which was purchased from CycLex Co., Ltd. (Cat#CY-1182). Absorbance was measured at 450 nm.

### Molecular Docking

**Figure 2:**
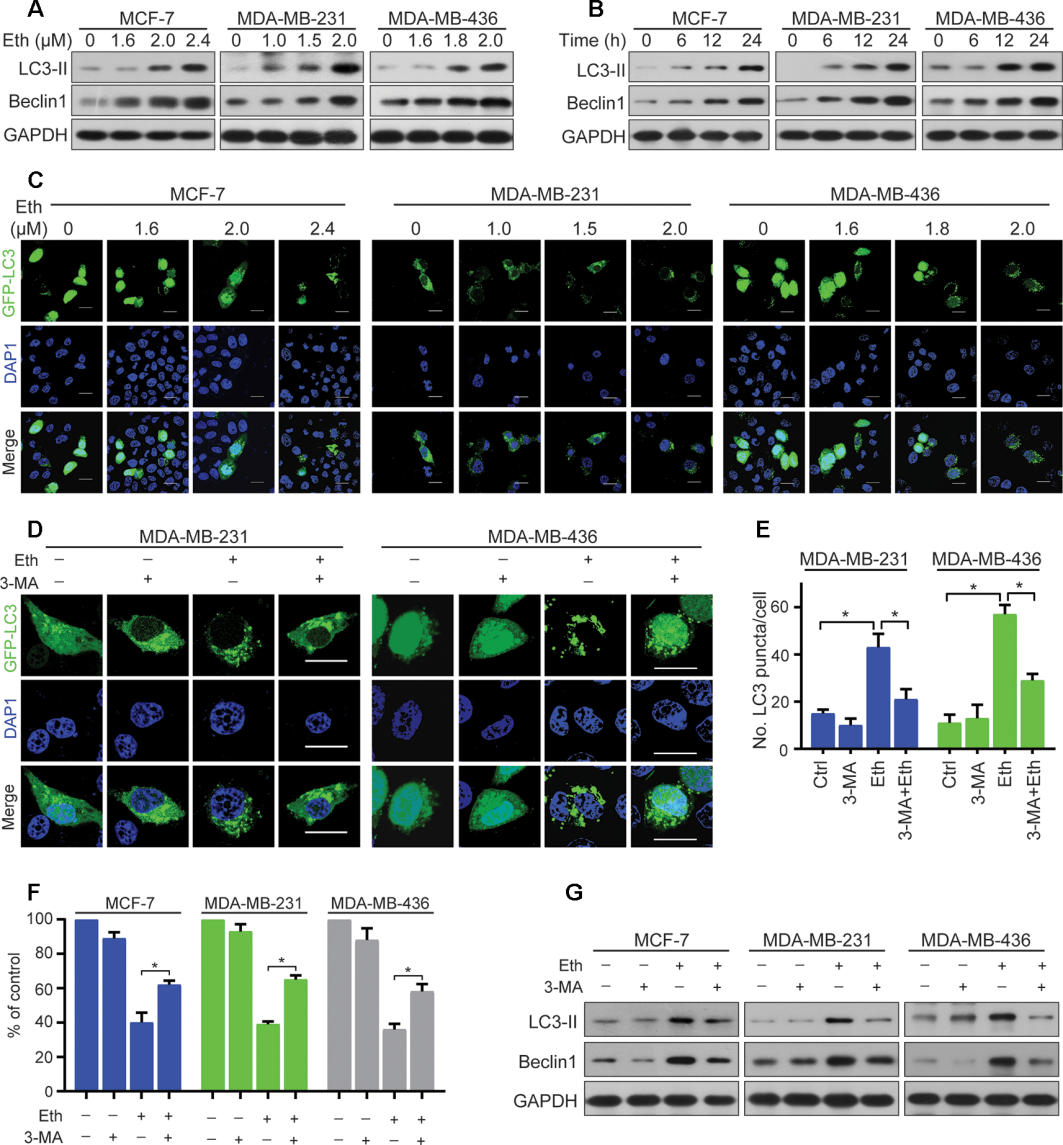
Eth induces autophagy in BC cells. **(A)**: MCF-7, MDA-MB-231, or MDA-MB-436 cells were treated with increasing concentrations of Eth for 24 h. Western blot was performed using antibodies indicated. GAPDH was used as the loading control. **(B)**: MCF-7 (MDA-MB-231 or MDA-MB-436) cells were treated with 2.4 µM (2.0 µM or 2.0 µM) Eth for the indicated times, and cell lysates were subjected to western blot assay. **(C)**: MCF-7, MDA-MB-231, or MDA-MB-436 cells transfected with pQCXIP-GFP-LC3 plasmid were treated with increasing concentrations of Eth for 24 h, and assessed by immunoﬂuorescence analyses. Scale bar = 20 µm. **(D)**: MDA-MB-231 or MDA-MB-436 cells transfected with pQCXIP-GFP-LC3 plasmid were treated with Eth (2.0 µM) and/or 3-MA (1 mM) for 24 h, and assessed by immunoﬂuorescence analyses. Scale bar = 20 µm. **(E)**: Graph shows quantification of LC3-positive punctate cells in **(D)**. **(F)**: MCF-7, MDA-MB-231, or MDA-MB-436 cells were treated with Eth (3.5 µM, 3.0 µM, or 3.0 µM) and/or 3-MA (1 mM) for 24 h and analyzed by MTT assay. **(G)**: MCF-7, MDA-MB-231, or MDA-MB-436 cells were treated with Eth (2.4 µM, 2.0 µM, or 2.0 µM) and/or 3-MA (1 mM) for 24 h, and western blot was performed using antibodies indicated. *P < 0.05.

Ligand docking studies were performed with Autodock4. The structure of AMPK (PDB code: 4CFF) was obtained from the protein data bank. α is green, β is cyan, γ is magenta, and the ligands are cyan. The chemical structure of Eth is shown in **Figure 2A**. Docking was performed with the docking box sizes large enough to include the binding sites.

### Immunoprecipitation and Streptavidin Agarose Affinity Assay

Cell pellets were lysed and the supernatant collection was incubated with indicated antibodies overnight at 4°C, after which protein A/G Plus beads (Santa Cruz Biotechnology) were added and incubated at 4°C for 4 h. The beads were washed 4 times in NETN buffer (1% NP-40, 2 mM EDTA, 40 mM Tris-HCl, 137 mM NaCl, pH 7.4), resuspended in SDS-PAGE loading buffer and boiled for 5 min. For streptavidin agarose affinity assay, cells upon Bio-Eth were lysed; the lysates were incubated with streptavidin agarose, washed and boiled in SDS-PAGE loading buffer. For Eth competition, the cell lysates were pretreated with Eth (4 μM) for 1 h, followed by 12 μM Bio-Eth treatment for 3 h at 4°C, and streptavidin agarose affinity assay were performed. Western blot assays were performed.

### Xenograft Studies

Female nude immunodeficient mice (nu/nu) (weighing ~16 g, 5-week-old) were purchased from Hunan SJA Laboratory Animal Co., Ltd. (Changsha, China), and maintained under specific pathogen-free conditions. Mice were given free access to sterile water and to a standard diet, and maintained under controlled conditions of temperature (22–24°C), humidity (40–70%), and light (12 h light-dark cycles). The present study was approved by Animal Ethics Committee of Hubei University of Medicine. The mice were subcutaneously injected with human BC MDA-MB-231 cells (5 × 10^6^ cells which were suspended in 100 μL DMEM) into the right flank of each mouse. Treatments started when tumors reached a palpable size (0.5 cm in diameter). Mice were randomly divided into the following two groups: control group (vehicle; 0.8% DMSO, 12% cremophor, and 8% ethanol in normal saline; n = 8) and Eth-treated group (intraperitoneal injection of 1 mg/kg Eth; n = 8). The mice were treated 5 times per week for a total of 30 weeks. Caliper measurements of the longest perpendicular tumor diameters were performed twice a week to estimate the tumor volume, using the following formula: 4π/3 × (width/2)^2^ × (length/2), representing the three-dimensional volume of an ellipse. Animals were sacrificed when tumors reached 1.5 cm or if the mice appeared moribund to prevent unnecessary morbidity to the mice.

### Pharmacokinetic Study

Eleven Sprague–Dawley rats (220–250 g, female) were bought from Hunan SJA Laboratory Animal Co., Ltd. (Changsha, China), and maintained and monitored in a specific pathogen-free environment. They were fasted overnight before the experiments. All rat studies were conducted according to protocols approved by the Animal Ethics Committee of Hubei University of Medicine. Six rats were administered with Eth (2 mg/kg) by intravenous injection, and the other five rats were inoculated with Eth (15 mg/kg) by intragastric injection. Blood samples of 100–200 mL were collected from the orbit at the time points indicated. The plasma concentrations of Eth were determined by nuclear magnetic resonance analysis (**Figure S1**). The pharmacokinetic parameters were obtained from the pharmacokinetic software DAS 2.0 (Drug and Statistics Version 2.0).

### Statistical Analysis

All experiments were repeated at least three times and the data are presented as the mean ± SD. All statistical analyses were conducted using GraphPad Prism 8 (GraphPad Software, Inc., La Jolla, CA, USA) and SPSS 22.0 (IBM Corp., Armonk, NY, USA). Results were analyzed using unpaired Student’s *t* test or one-way analysis of variance followed by Bonferroni post-test. *P* < 0.05 was considered to indicate a statistically significant difference.

## Results

### Effects of Eth on BC Cells

The effect of Eth on cell growth was investigated with seven BC cell lines, MCF-7, SK-BR3, MDA-MB-231, MDA-MB-436, MDA-MB-468, MDA-MB-453, and MDA-MB-435S. Using an MTT assay, we found that Eth had moderate cytotoxicity to these cell lines, with IC_50_ values ranging from 2.63 μM to 9.15 μM (**Figure 1C**, **Table 1**). The normal human mammary epithelial cell line MCF-10A was less sensitive to Eth than the BC cell lines. As shown in **Figures 1D–F**, Eth was effective in inhibiting the growth of MCF-7, MDA-MB-231, and MDA-MB-436 BC cells. Using a trypan blue exclusion assay, we found that Eth rapidly reduced viable MCF-7 (**Figure 1G**), MDA-MB-231 (**Figure 1H**), and MDA-MB-436 cells (**Figure 1I**) in a dose- and time-dependent manner. We investigated the effect of Eth on cell colony formation activity, and the results showed that Eth significantly inhibited the clonogenic ability of MCF-7, MDA-MB-231, and MDA-MB-436 cells (**Figures 1J, K**). These results suggested that Eth inhibited the anchorage-dependent (cell proliferation) and anchorage-independent (colony formation) growth of BC cells.

**Table 1:** IC_50_s of Eth on BC cell lines.

Cell lines	MCF-10A	T47D	MDA-MB-453	MDA-MB-468	MDA-MB-435S	MDA-MB-231	MCF-7	SK-BR3	MDA-MB-436
**IC_50_**	12.30 ± 2.05	9.15 ± 1.83	7.06 ± 0.59	6.67 ± 1.70	4.58 ± 0.77	3.75 ± 0.94	3.29 ± 1.50	2.79 ± 0.39	2.63 ± 0.65

The cells were treated with Eth at various concentrations for 24 h, the cell cytotoxicity was analyzed by MTT assay, and the IC_50_ was calculated using CalcuSyn (version 2.1). Values shown are means plus or minus SD of quadruplicate determinations.

### Eth Induces Autophagy in BC Cells

Autophagy is the process of sequestrating cytoplasmic proteins into lytic compartments and is characterized by the formation of the autophagosome (LC3-positive vesicle), a double membraned structure that sequesters the target organelle/protein and then fuses with endo/lysosomes where the contents are its major component (Kong et al., 2017).We tested whether Eth could induce autophagy in BC cells by detecting changes in the lipidated form (LC3-II) of the autophagy marker LC3. Interestingly, we found that Eth induced the accumulation of LC3-II and Beclin-1in MCF-7, MDA-MB-231, and MDA-MB-436 cells in a dose- and time-dependent fashion (**Figures 2A**, **B**). Accordingly, the pQCXIP-GFP-LC3 plasmid was transfected into MCF-7, MDA-MB-231, and MDA-MB-436 cells which were then treated with Eth for 24 h, followed by confocal microscopy assessment. We showed that while control cells displayed diffuse staining, MCF-7, MDA-MB-231, and MDA-MB-436 cells upon Eth exhibited a speckled fluorescent staining pattern, indicating the redistribution of LC3 to autophagosomes (**Figure 2C**). Autophagy has been reported to play contradictory roles in tumor progression and suppression (Lin and Baehrecke, 2015). To demonstrate Eth induced autophagy, Eth and the autophagy inhibitor 3-MA were combined to treat MCF-7, MDA-MB-231, and MDA-MB-436 cells. Our results showed that 3-MA could antagonize the induction of autophagy by Eth (**Figures 2D**, **E**). 3-MA significantly reversed the cell proliferation inhibited by Eth (**Figure 2F**). Furthermore, 3-MA antagonized the upregulation of autophagy-related proteins LC-3II and Beclin1 by Eth (**Figure 2G**). Taken together, the data presented here indicate that Eth induces autophagy in BC cells.

### Eth Induces Autophagy Through AMPK/mTORC1 Signaling

**Figure 3:**
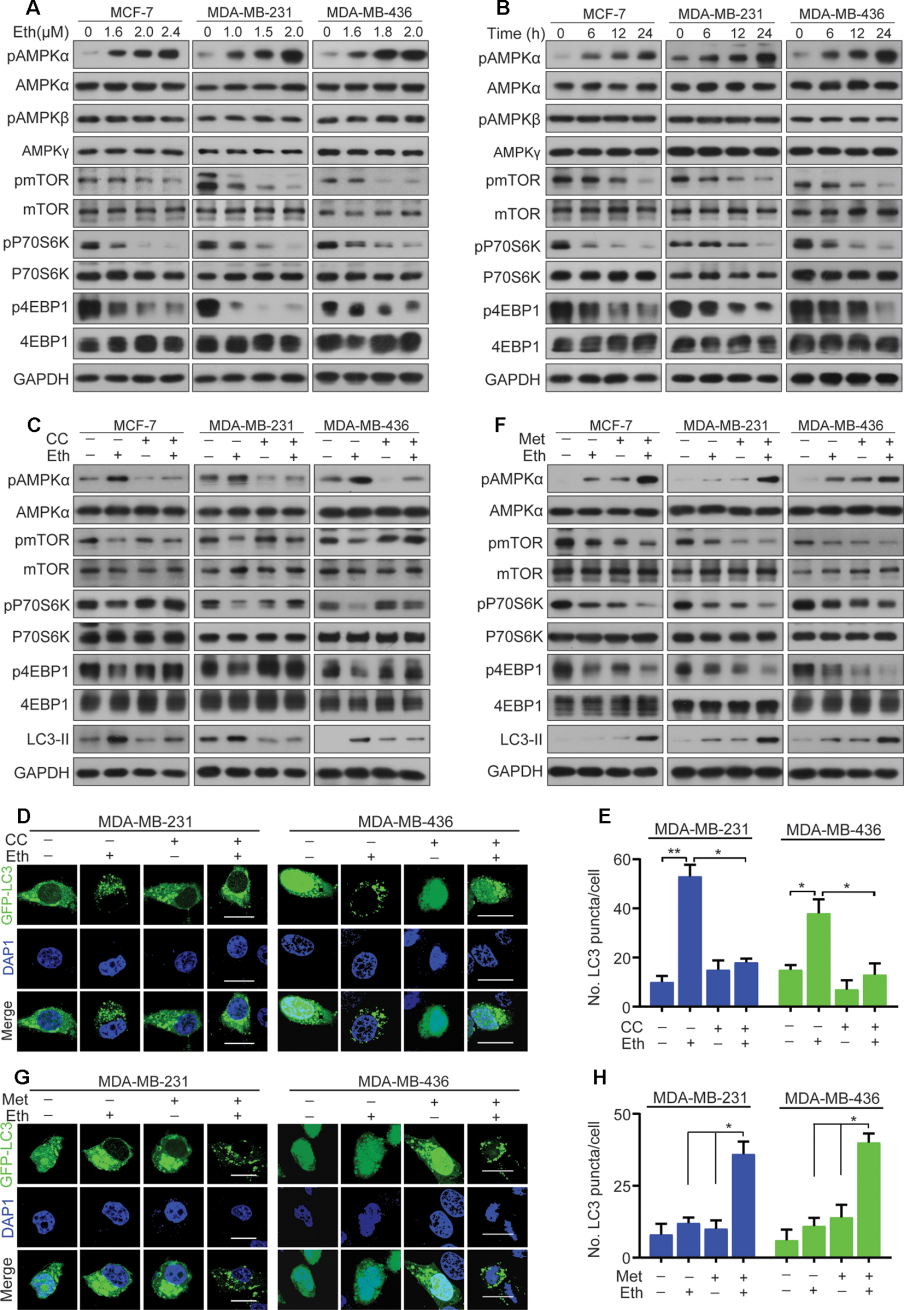
Eth induces autophagy through the AMPK/mTORC1 signaling. **(A)**: MCF-7, MDA-MB-231, or MDA-MB-436 cells were treated with increasing concentrations of Eth for 24 h. Western blot was performed using antibodies indicated. **(B)**: MCF-7, MDA-MB-231, or MDA-MB-436 cells were treated with 2.4 µM, 2.0 µM, or 2.0 µM Eth for the indicated times, and cell lysates were subjected to western blot assay. **(C)**: MCF-7, MDA-MB-231, or MDA-MB-436 cells were treated with Eth (2.4 µM, 2.0 µM, or 2.0 µM) and/or Compound C (CC, 25 µM) for 24 h, and western blot was performed using antibodies indicated. **(D)**: MDA-MB-231 or MDA-MB-436 cells transfected with pQCXIP-GFP-LC3 plasmid were treated with Eth (2.0 µM) and/or CC (25 µM) for 24 h, and assessed by immunoﬂuorescence analyses. Scale bar = 20 µm. **(E)**: Graph shows quantification of LC3-positive punctate cells in **(D)**. **(F)**: MCF-7, MDA-MB-231, or MDA-MB-436 cells were treated with Eth (1.6 µM, 1.0 µM, or 1.6 µM) and/or metformin (Met, 10 mM) for 24 h, and western blot was performed using antibodies indicated. **(G)**: MDA-MB-231 or MDA-MB-436 cells transfected with pQCXIP-GFP-LC3 plasmid were treated with Eth (1.0 µM or 1.6 µM) and/or Met (10 mM) for 24 h, and assessed by immunoﬂuorescence analyses. Scale bar = 20 µm. **(H)**: Graph shows quantification of LC3-positive punctate cells in **(G)**. *P < 0.05, **P < 0.01.

Next, we examined the target signal pathway of Eth inducing autophagy in BC cells. A pivotal role in the control of autophagy is played by mTORC1 (Rabanal-Ruiz et al., 2017), which brings together regulatory information from multiple upstream signal transduction pathways, including AMPK. AMPK is induced by various conditions of stress that are known to activate autophagy (Wang et al., 2016). The AMPK subunits were therefore determined to define their roles in BC cells treated with Eth. As shown in **Figures 3A**, **B**, the phosphorylation of AMPKα at Thr172, instead of the β- or γ-subunits, was increased upon Eth treatment in both a dose- and time-dependent manner. We next detected mTORC1, the downstream targets of AMPK, and found that Eth decreased the phosphorylation of mTORC1 effectors (mTOR, p70S6K, and 4EBP1) in an AMPK dependent manner. Based on the above results, we hypothesized that Eth inhibits cell proliferation and induces autophagy depending on AMPK activation. To test this hypothesis, we treated MCF-7, MDA-MB-231, and MDA-MB-436 cells with Eth alone and in combination with compound C (CC), an AMPK inhibitor. Our data showed that a significant inhibition of the phosphorylation of mTORC1 was observed in cells treated with Eth alone. However, treatment with CC reversed the reduction in phosphorylation (**Figure 3C**), and reversed the increased LC-3II expression and number of autophagic vacuoles in Eth-treated cells (**Figures 3C–E**). Moreover, co-treatment of Eth and metformin, a known AMPK activator, enhanced the effect of Eth on the phosphorylation of mTORC1 and autophagy (**Figures 3F–H**). Altogether, these findings indicate that Eth induces autophagy through the AMPK/mTORC1 signaling.

### Eth Is a Direct Activator of AMPK

**Figure 4:**
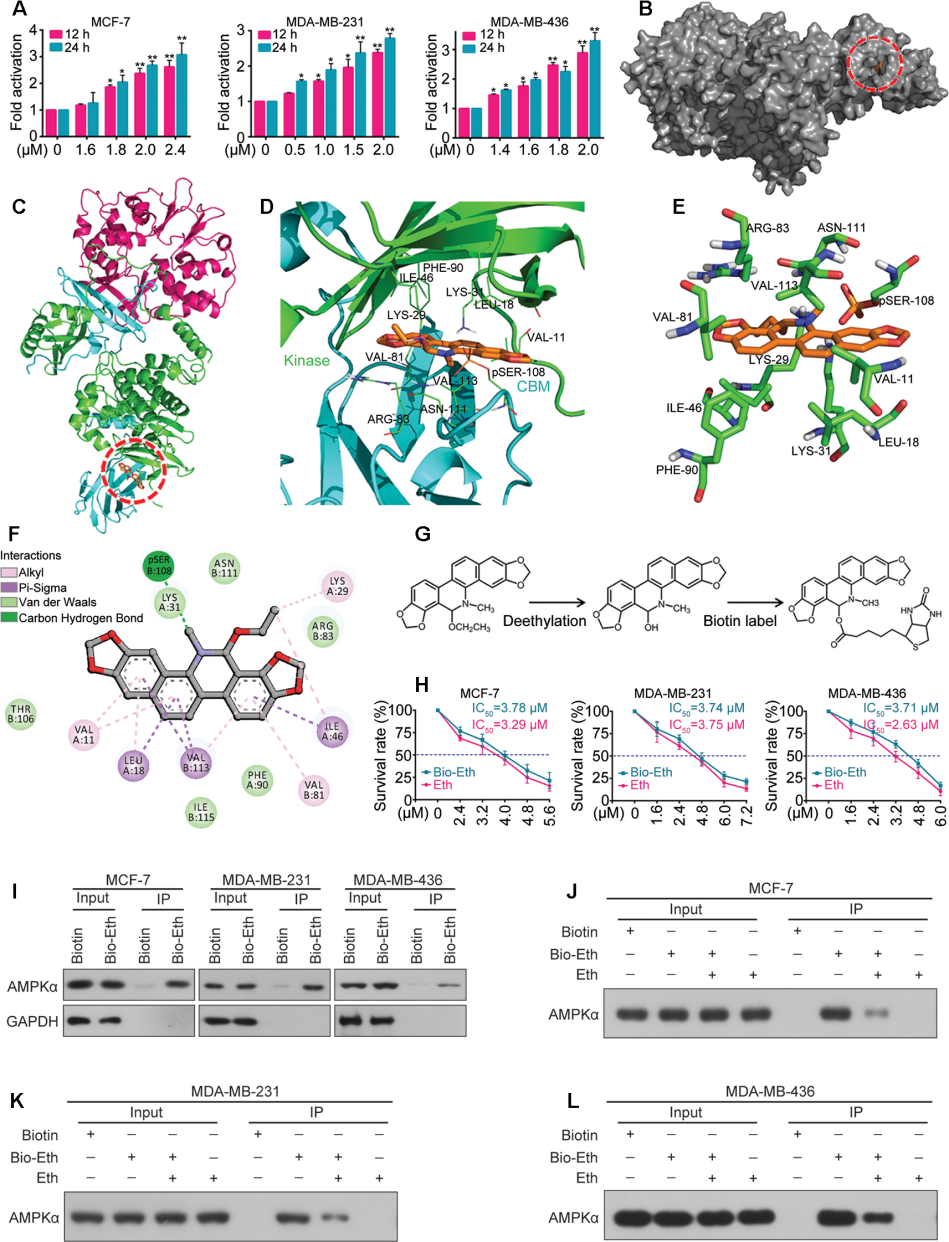
Eth is a direct activator of AMPK. **(A)**: MCF-7, MDA-MB-231, or MDA-MB-436 cells were treated with increasing concentrations of Eth for 24 h, and AMPK activity assay was performed. **(B)**: The binding mode of Eth docked into AMPK. **(C)**: The binding mode of Eth docked into AMPK. α subunit is green, β subunit is cyan, γ subunit is magenta and Eth is gold. **(D)**: Details of the binding site of Eth in the ADaM site of AMPK. The main residues involved in drug interactions are shown as labeled green sticks. α subunit is green, β subunit is cyan. The pSER-108 was shown in stick and colored by gold. **(E)** The interaction pattern of Eth with the residues. **(F)**: The 2D representation of the AMPK crystal structure in complex with Eth. **(G)**: Chemical structure of Eth intermediate and Biotin-labeled Eth. **(H)**: Viability of MCF-7, MDA-MB-231 or MDA-MB-436 cells exposed to Biotin-Eth as determined by MTT assay. **(I)**: MCF-7, MDA-MB-231, or MDA-MB-436 cells were treated with 4 μM Biotin or Bio-Eth for 12 h, lysed, and the cell lysates were subjected to immunoprecipitation using S. agarose and western blot using indicated antibodies. **(J**–**L)**: MCF-7, MDA-MB-231, or MDA-MB-436 cells were treated with Bio-Eth (4 μM) in the presence or absence of Eth (12 μM) for 3 h, lysed, and the cell lysates were subjected to immunoprecipitation and western blot. *P < 0.05, **P < 0.01.

Next we determined whether Eth modulates the activity of the AMPK kinase. For this purpose, MCF-7, MDA-MB-231, and MDA-MB-436 cells were treated with Eth and incubated for different time periods (1, 6, 12, and 24 h). The AMPK activity assay revealed a robust increase in AMPK activity after Eth treatment in the three cell lines (**Figure 4A**). These results confirmed the potential of Eth as a potent AMPK activator.

We further determined the interaction of Eth and AMPK *in silico*. Molecular docking experiments were conducted between Eth and the crystal structure of AMPK (PDB code: 4CFF) by Autodock4. The greater the negative energy was, the larger the sum of the physical terms that present the combined free energy. The lower the energy was, the better the docking orientation. Some orientations had similar poses of similar energy and were considered a set of hits. Among the α, β, and γ subunits, there is an ADaM site (allosteric drug and metabolite site), which was constructed by the catalytic kinase domain of α subunit and the regulatory carbohydrate-binding module (CBM) of the β subunit. Eth bound to the ADaM site with a binding energy of –7.4 Kcal/Mol (**Figures 4B**, **C**) and interacted with a cluster of hydrophobic residues from each domain: VAL-11_(Kinase)_, LEU-18_(Kinase)_, LYS-29_(Kinase)_, LYS-31_(Kinase)_, ILE-46_(Kinase)_, PHE-90_(Kinase)_, VAL-81_(CBM)_, ARG-83_(CBM)_, SER-108_(CBM)_, ASN-111_(CBM)_, and Val-113_(CBM)_ (**Figures 4D–F**). To confirm the Eth/AMPK interaction, we first generated biotinylated Eth. Eth does not contain modifiable sites such as hydroxyl (–OH) and carboxyl (–COOH) sites; thus, we removed the ethyl in the ethoxy group and linked biotin to obtain biotinylated Eth (Bio-Eth, **Figure 4G**). To ensure that the generated Bio-Eth retained its inhibitory activity in BC, we assessed cell viability and compared the results to Eth. we found that the IC_50_ values of Bio-Eth in the MCF-7, MDA-MB-231, and MDA-MB-436 cells were 3.78 μM, 3.74 μM, and 3.71 μM, respectively, which were more similar to the IC_50_ values (3.29 μM, 3.75 μM, and 2.63 μM, Respectively) of Eth (**Figure 4H**). Therefore, Bio-Eth was selected for subsequent study. In Bio-Eth-treated MCF-7, MDA-MB-231, and MDA-MB-436 Cells, AMPKα was pulled down by streptavidin agarose (**Figure 4I**). The *in Vitro* experiment showed that the binding of Bio-Eth to AMPKα was significantly attenuated by unlabeled Eth (**Figures 4J–L**), indicating the direct binding of Eth to AMPK.

### Eth Impairs Glucose Metabolism in BC Cells

AMPK is a major regulator of cellular homeostasis and is activated in response to metabolic stress. We investigated the effect of Eth on energy metabolism (Hardie and Lin, 2017). We detected the expression and activity of acetyl-CoA carboxylase (ACC), which is an intermediate substrate playing a pivotal role in the regulation of fatty acid metabolism and energy production, and found Eth has no significant effect on the expression and activation of ACC. We detected the expression of glucose and lactate metabolism-associated proteins and found that Eth downregulated the expression of glucose uptake-associated proteins Glut1, Glut3, and Glut4 (**Figures S2** and **S3**), suggesting that Eth significantly impairs glucose metabolism in BC cells.

### AMPKα Is Phosphorylated at Low Levels in BC and Correlates With Clinicopathological Parameters

**Figure 5:**
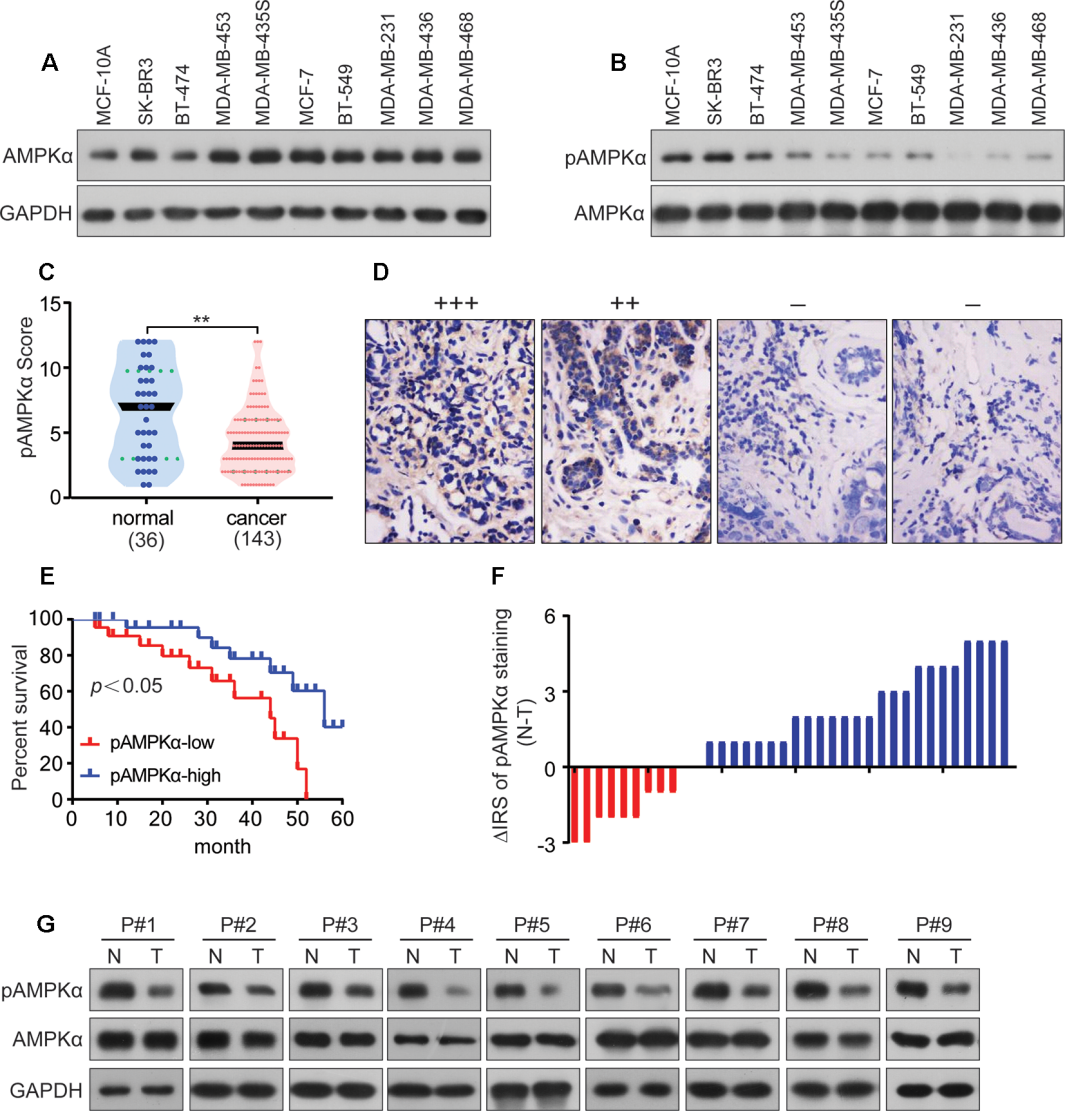
AMPKα is low phosphorylated in BC and correlates with clinicopathological parameters. **(A**, **B)**: Western blot analysis showed AMPKα expression and phosphorylation in different cell lines. **(C)**: Immunohistochemistry analysis of AMPKα phosphorylation levels in BC specimens and normal tissues. **P < 0.01. **(D)**: Representative immunohistochemical staining examples of AMPKα phosphorylation in BC tissues. **(E)**: Survival curves of BC patients with low phosphorylation versus high phosphorylation of AMPKα. (F): The distribution of the difference in AMPKα phosphorylation staining (ΔIRS = IRSN−IRST). Immunoreactivity score (IRS) of AMPKα phosphorylation staining was available from 36 pairs of tissues; *P* values were calculated with the Wilcoxon test. AMPKα phosphorylation was lower in tumor tissues (T) compared with paired adjacent non-tumor tissues (N). IRST, IRS of tumor tissues; IRSN, IRS of non-tumor tissues. *P* < 0.05. **(G)**: The expression and phosphorylation of AMPKα in 9 human BC tumor tissues and adjacent non-tumor tissues (N) as tested by western blot assay.

AMPKα expression and phosphorylation (pAMPKα) were studied by western blot in the BC cell lines MCF-7, BT-474, SK-BR3, BT-549, MDA-MB-453, MDA-MB-435S, MDA-MB-468, MDA-MB-231, and MDA-MB-436, and the normal human mammary epithelial cell line MCF-10A. The results showed that AMPKα was phosphorylated at low levels in most of the investigated BC cell lines (MCF-7, MDA-MB-468, MDA-MB-231, and MDA-MB-436) (**Figures 5A**, **B**). We also analyzed pAMPKα expression in 143 BC patients specimens and 36 adjacent normal tissues from Hubei Province in central China using immunohistochemistry analysis. We found that pAMPKα was decreased in 52% of the tumor samples (75 of 143), and the adjacent normal tissues exhibited high (or moderate) pAMPKα staining (26 of 36, 72%) (**Figures 5C**, **D**). These results suggested that AMPKα phosphorylation might be a critical mechanism in BC development. The clinical characteristics of the patients are shown in **Table 2**. No significant correlation was observed between AMPKα phosphorylation and age, tumor size, or TNM stage (*P* > 0.05). However, significant correlations between AMPKα phosphorylation and grade and distant metastasis were observed (*P*< 0.05). To further determine the relationship between AMPKα phosphorylation and the survival of BC patients, we performed Kaplan-Meier analysis based on the available follow-up data from 38 BC patients. The survival analysis revealed that a survival advantage was identified in patients whose tumors had higher AMPKα phosphorylation than those with lower AMPKα phosphorylation (**Figure 5E**, *P* < 0.05), indicating that lower AMPKα phosphorylation is related to a poor clinical outcome in BC. In 36 cases of BC tissues with paired adjacent nontumor tissues, we observed a significantly lower phosphorylation of AMPKα in tumor tissues compared with the paired adjacent nontumor tissues (*P* < 0.05, **Figure 5F**). Western blot analyses were used to detect the phosphorylation of AMPKα in the human BC tissues of 9 patients. AMPKα exhibited lower phosphorylation in all tumor tissues compared with the patient-matched adjacent normal colonic tissues (**Figure 5G**). These results indicated that AMPK phosphorylation might be a critical event in BC development.

**Table 2:** Characteristics of AMPKα phosphorylation in BC patients.

Characteristics	AMPKα phosphorylation		*P* value^†^
	low (n = 75)	high (n = 68)	
	No.	No.	
**Age**			
<45	23	21	0.56
≧45	52	47	
**Tumor size**			
<5 cm	49	40	0.27
≧5 cm	26	28	
**TNM stage**			
I-II	30	37	0.06
III-IV	45	31	
**Grade**			
1–2	32	40	0.04
3	43	28
**Distant metastasis**			
Negative	29	38	0.03
Positive	46	30	

^†^Chi-square test.

### Eth Inhibits Tumor Growth in Murine Models and Shows Favorable Pharmacokinetic Profiles

**Figure 6:**
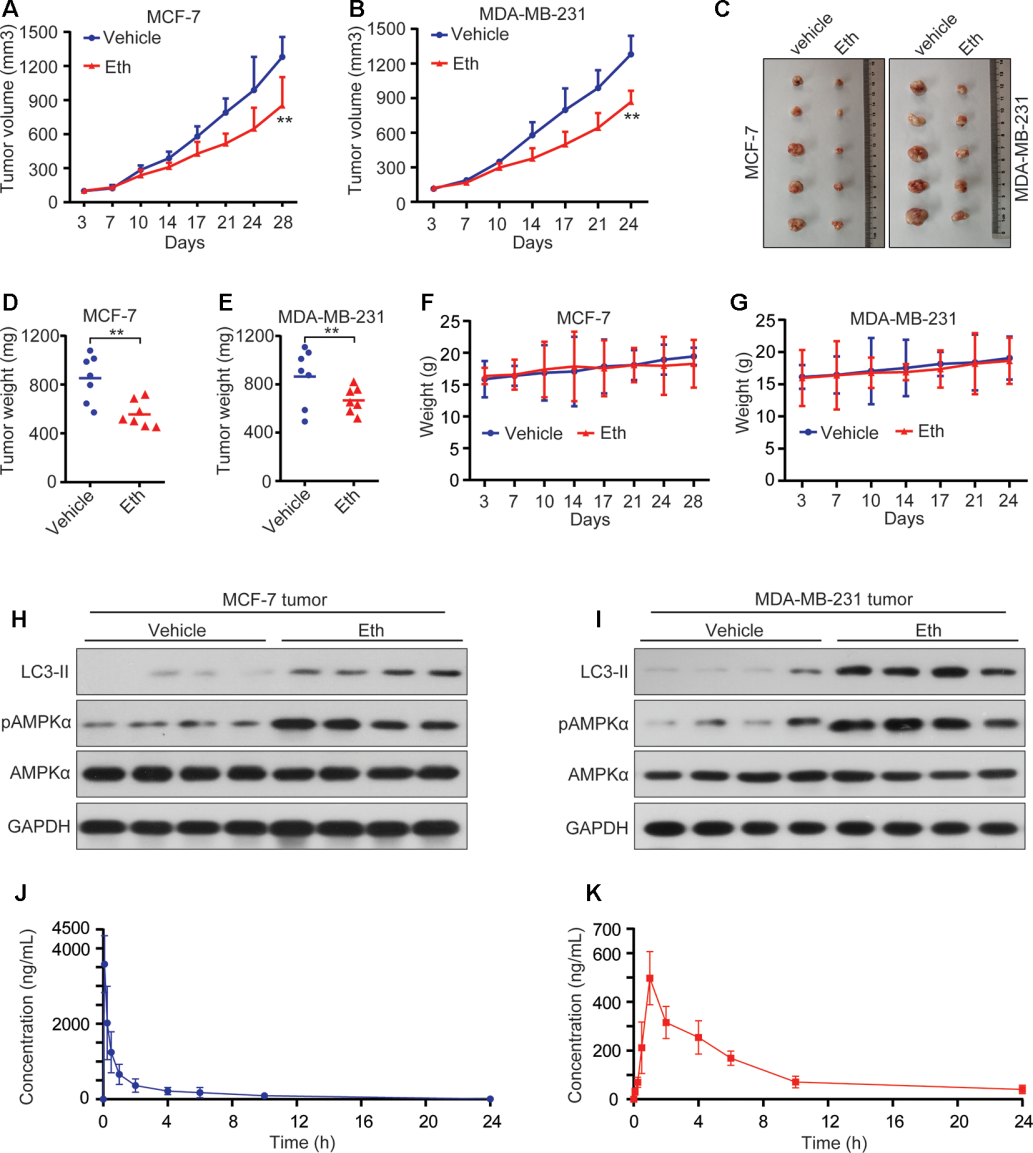
Eth inhibits tumor growth in murine models and shows favorable pharmacokinetic profiles. **(A**–**B)**: Murine models were treated with vehicle, Eth (2 mg/kg) and the tumor volumes were calculated twice a week. **P < 0.01. **(C)**: Images of xenograft tumors obtained from mice with different treatment after about 4 weeks. **(D**–**E)**: Weight of the tumor from each group taken out from the sacrificed mice at the end of the study **P < 0.01. **(F**–**G)** Eth treatment did not affect the murine model body weight. **(H**–**I)**: The expressions of LC3-II, AMPKα, and pAMPKα in xenograft tumor tissues were analyzed by western blot. **(J)**: The concentration-time profiles of Eth after *i.v.* injection (2 mg/kg) in Sprague-Dawley rats. **(K)**: The concentration-time profiles of Eth after *i.g.* injection (15 mg/kg) in Sprague-Dawley rats.

To determine the anti-tumor effect of Eth on BC *in vivo*, MCF-7 xenografted and MDA-MB-231 xenografted murine models were generated. Once the tumors grew to a measurable size, each group was administered with vehicle (MCF-7 = 8, MDA-MB-231 = 8) or Eth (MCF-7 = 8; MDA-MB-231 = 8) *i.p.* at 2 mg/kg four times a week for 4 weeks. The tumor-bearing mice were humanely killed when their tumors reached 1.5 cm in diameter or when paralysis or major compromise in their quality of life occurred. We found that Eth efficiently repressed tumor growth compared with the vehicle control (*P* < 0.05; **Figures 6A–C**). Eth treatment also significantly reduced the tumor weight of the mice (**Figures 6D**, **E**). In addition, Eth treatment did not significantly reduce the body weight of the mice. This finding suggested that Eth did not cause evident side effects (**Figures 6F**, **G**). All of the mice were euthanized, and the tumor specimens were examined by western blot. The results showed that the expression levels of pAMPK and LC-3II were upregulated in the Eth-treated groups (**Figure 6E**).

We then tested the pharmacokinetic features of Eth in Sprague-Dawley rats. To do this, six rats were administered Eth (2 mg/kg) by intravenous (*i.v.*) injection, and another five rats were inoculated with Eth (15 mg/kg) by intragastric (*i.g.*) injection. the mean plasma concentration-time profiles are shown in **Figures 6J**, **K**, and the main pharmacokinetic parameters are summarized in **Table 3**. We found that in the six rats that received an *i.v.* injection of Eth At 2 mg/kg, the Eth in plasma achieved a peak concentration of 3.578 ± 0.756 mg/L At 0.083 h (1.980 min), with a T_1/2_ of 3.539 h in five rats administrated Eth at 15 mg/kg *i.g.* injection, the Eth in plasma achieved a peak concentration of 0.498 ± 0.110 mg/L at 2.000 h, with a T_1/2_ of 4.049 h. These results demonstrate that the administration of Eth *via i.v.* injection can reach the therapeutic concentration of Eth used *in vivo*. Therefore, Eth could be a potential therapeutic agent against BC.

**Table 3:** Pharmacokinetic parameters of Eth in Sprague-Dawley rats.

Administration and dosage	T_1/2_ (h)	AUC_0-t_ (mg/L·h)	T_max_ (h)	C_max_ (mg/L)	MRT_0-t_ (h)	CL_z_ (L/h/kg)	BA (%)
*i.v.* (2 mg/kg)	3.539 ± 1.790	3.639 ± 1.555	0.083 ± 0.000	3.578 ± 0.756	2.178 ± 0.462	0.559 ± 0.197	—
*i.g.* (15 mg/kg)	4.049 ± 1.578	3.392 ± 0.592	2.0 ± 0.000	0.498 ± 0.110	7.031 ± 1.014	4.418 ± 0.892	12.43

AUC, Area under the plasma concentration time curve; BA, bioavailability; CL, total plasma clearance; C_max_, maximum observed plasma concentration; i.v., intravenous injection; i.g., intragastric injection; MRT, mean residence time; T_1/2_, terminal elimination phase half-life.

## Discussion

Traditional Chinese medicine, as an important source of medicine and therapeutics, plays a critical role in the treatment of numerous human diseases. Eth, an active natural compound, has been reported to possess potential anticancer activities by targeting the oncoprotein CIP2A (Liu et al., 2014; Jin et al., 2018). Here, we have demonstrated for the first time that Eth induces the activation of autophagy as a new direct AMPK activator, resulting in the inhibition of BC proliferation *in vitro* and *in vivo*. The different mechanisms of action of Eth in different tumors suggest that Eth may be a multi-targeted drug, or that a regulatory mechanism exists between CIP2A and AMPK.

However, the role of autophagy in cancer treatment is still controversial. On the one hand, autophagy may promote cell death (Denton and Kumar, 2018; Kriel and Loos, 2019); on the other hand, autophagy may play a supporting role in the malignant progression and drug resistance of tumors (Li et al., 2017; Mowers et al., 2017). In this study, we demonstrated that Eth can induce autophagosome biosynthesis, manifested by increased autophagosome formation, LC3-II conversion, and LC3B-puncta (**Figures 2A–C**). 3-MA specifically blocks autophagosome formation and is widely used as an autophagy inhibitor. We further found that 3-MA can partially block the activation of autophagy by Eth and further block the inhibition of Eth on cell proliferation (**Figures 2D–G**), suggesting that Eth functions as a promising antitumor agent by inducing a particular autophagy-activating effect.

As one of the main metabolic sensors, AMPK plays a pivotal role in the regulation of the autophagy process. Conventionally, AMPK triggers autophagy by directly activating unc-51 like autophagy activating kinase 1 (ULK1) or indirectly inhibiting mTORC1, both of which ultimately lead to autophagosome aggregation and autophagy activation by Beclin1 (Wang et al., 2016; Hardie and Lin, 2017). Here, we demonstrated that Eth significantly induced the phosphorylation of AMPK and inactivation of the mTORC1 complex to elicit autophagy (**Figures 3A**, **B**). Further studies have found that the AMPK inhibitor CC can antagonize the inhibitory effect of Eth on mTORC1 signaling and the activation of autophagy (**Figures 3C–E**). At the same time, we co-treated Eth and AMPK activator metformin and found that they synergistically inhibited mTORC1 signaling and the synergistic activation of autophagy (**Figures 3F–H**). Based on the results above, we hypothesized that the inhibition of mTORC1 and induction of autophagy by Eth was dependent on AMPK activation. By using AMPK enzyme activity assay, we confirmed that Eth had a remarkable AMPK activation ability (**Figure 4A**). *In silico* modeling, we employed to investigate the potential interactions between Eth and AMPK. The results indicated that the ADaM active site of AMPK was the preferred binding site for Eth (**Figures 4B**, **C**). The ADaM site has been used to design selective activators of AMPK containing the β1 subunit and to screen β1-selective AMPK activators (Huang et al., 2017). Several direct AMPK activators (A-769662 (Shin et al., 2014), Salicylate (Liao et al., 2014), and Compound 991 (Bultot et al., 2016)) bound to the ADaM site and showed better potency. These activators activate AMPK both directly and through increased protection against T172 dephosphorylation (Yan et al., 2018). Eth interacted with a cluster of hydrophobic residues (VAL-11_(Kinase)_, LEU-18_(Kinase)_, LYS-29_(Kinase)_, LYS-31_(Kinase)_, ILE-46_(Kinase)_, PHE-90_(Kinase)_, VAL-81_(CBM)_, ARG-83_(CBM)_, SER-108_(CBM)_, ASN-111_(CBM)_, and VALl-113_(CBM)_) in the ADaM site, especially the hydrogen bond formed with phosphorylated serine (pSER-108). SER-108 is an autophosphorylation site on the β1 subunit, and the sustained phosphorylation of this site is beneficial for maintaining the stability of the ADaM site and ligand binding (Huang et al., 2017). Eth induces AMPK α, β subunit allosteric change by its benzene ring, methyl group, and ethoxy group through transient weak interactions such as Pi-sigma force, Van der waals and carbon hydrogen bond. Phosphorylation of Thr172 on the AMPK α subunit is then maintained to maintain AMPK activity. Furthermore, the Bio-Eth/streptavidin-agarose pull-down experiment demonstrated the direct interaction of Eth/AMPK (**Figures 4G–L**). AMPK acts as tumor suppressor to inhibit carcinogenesis, and promote apoptosis (Sun and Zhu, 2017). We showed that in 75/143 (52%) BCs, pAMPK was lower in tumor samples than in normal breast tissues (**Figures 5C**, **D**). Moreover, low AMPK phosphorylation was inversely associated with the poor prognosis of the patients (**Figure 5E**). These results suggest that AMPK activation may have a critical role in breast carcinogenesis. In xenografted murine models for BC, Eth significantly inhibited tumor growth (**Figures 6A–E**), and activated autophagy and AMPK *in vivo* (**Figures 6H**, **I**). Moreover, Eth exhibited favorable pharmacokinetics in rats (**Figures 6J**, **K**).

In summary, our study has provided a new compound structure, Eth, which directly binds to the ADaM site of AMPK and activates AMPK, exhibiting an anticancer effect on BC cells. It induced autophagy through AMPK/mTORC1 signaling to inhibit cancer cell proliferation. Our results suggest that Eth is a potential agent for treating BC patients and a promising drug for AMPK research.

## Data Availability Statement

The raw data supporting the conclusions of this article will be made available by the authors, without undue reservation, to any qualified researcher.

## Ethics Statement

This study was carried out in accordance with the recommendations of the Institutional Review Board of Hubei University of Medicine with written informed consent from all subjects. All subjects gave written informed consent in accordance with the Declaration of Helsinki. The protocol was approved by the Ethics Committee of Hubei University of Medicine. This study was carried out in accordance with the recommendations of the rules of Regulations for the Administration of Affairs Concerning Experimental Animals, Hubei University of Medicine Animal Care and Use Committee. The protocol was approved by the Hubei University of Medicine Animal Care and Use Committee.

## Author Contributions

YS, JW, and XL performed most of the experiments. ToZ, YX, TeZ, XW, TF, LX, QY, and HZ performed some of the experiments. XW provided some of the reagents and advice. YL designed the project and wrote the main manuscript text. All authors reviewed the manuscript.

## Funding

This work was supported by grants from the National Natural Science Foundation of China (no. 81802387), the Foundation for Innovative Research Team of Hubei Provincial Department of Education (no. T201915), the Natural Science Foundation of Hubei Province of China (no. 2018CFB586), the Principal Investigator Grant of Hubei University of Medicine (no. HBMUPI201806), the Faculty Development Grants from Hubei University of Medicine (nos. 2018QDJZR03 and 2018QDJZR27), the Innovative Research Program for Graduates (no. YC2019001), the Scientific and Technological Project of Shiyan City of Hubei Province (nos. 18Y13 and 19Y15) and Hubei Provincial Technology Innovation Project (no. 2017ACA176).

## Conflict of Interest

The authors declare that the research was conducted in the absence of any commercial or financial relationships that could be construed as a potential conflict of interest.

## Supplementary Material

Figure S1 Nuclear magnetic resonance analysis of Bio-Eth.

Figure S2 Eth impairs glucose metabolism in BC cells MCF-7 or MDA-MB-231cells were treated with increasing concentrations of Eth for 24 h. Western blot wasperformed using antibodies indicated.

Figure S3 Uncropped immunoblot for some protein.
