# Supplementary figures and images for: Ethoxysanguinarine, a Novel Direct Activator of AMP-Activated Protein Kinase, Induces Autophagy and Exhibits Therapeutic Potential in Breast Cancer Cells
Source: Front Pharmacol. 2020 Jan 8;10:1503. doi: 10.3389/fphar.2019.01503 (PMC6960228; doi:10.3389/fphar.2019.01503)

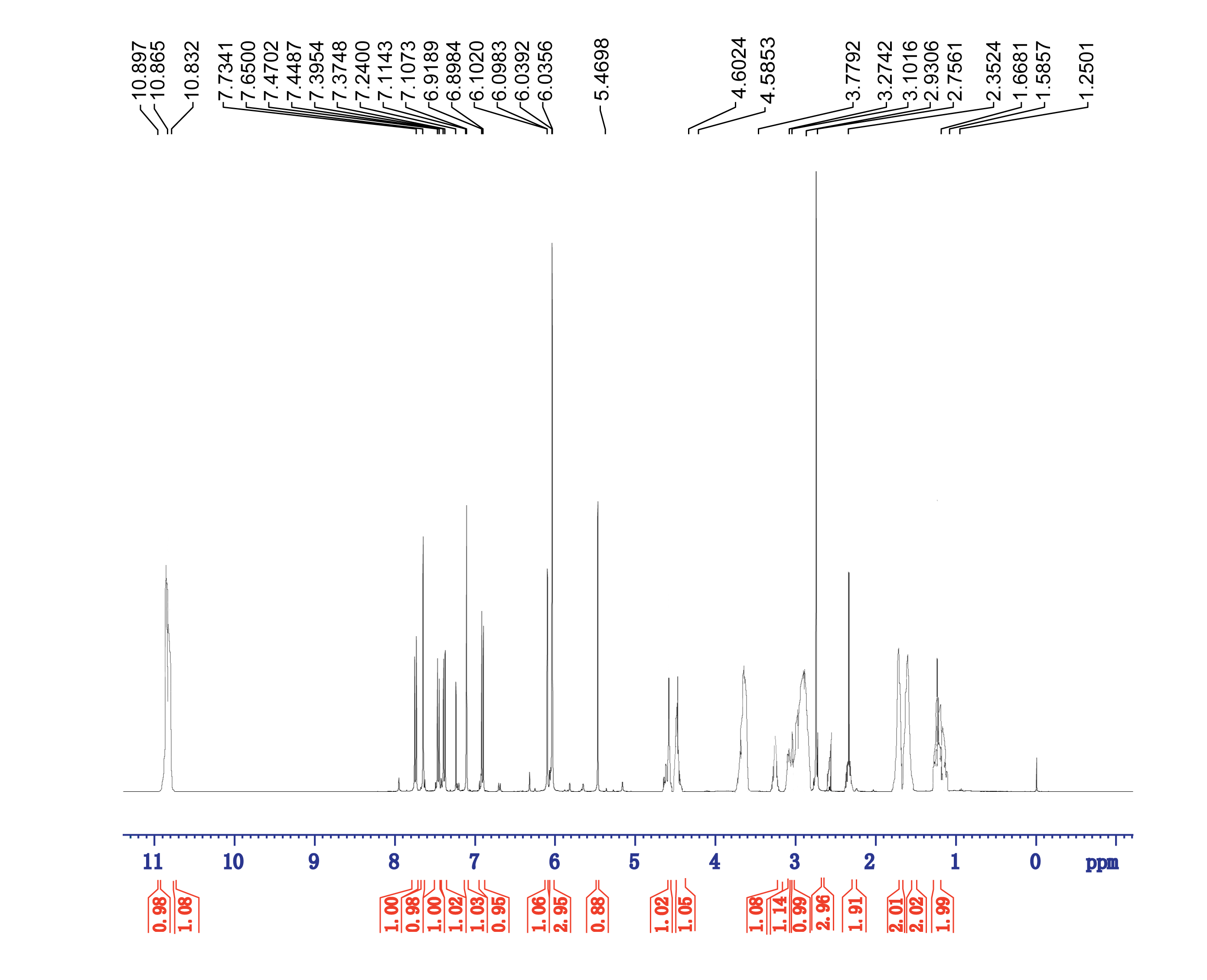

Supplement: Figure S1 — Nuclear magnetic resonance analysis of Bio-Eth. [file Image_1.tif]

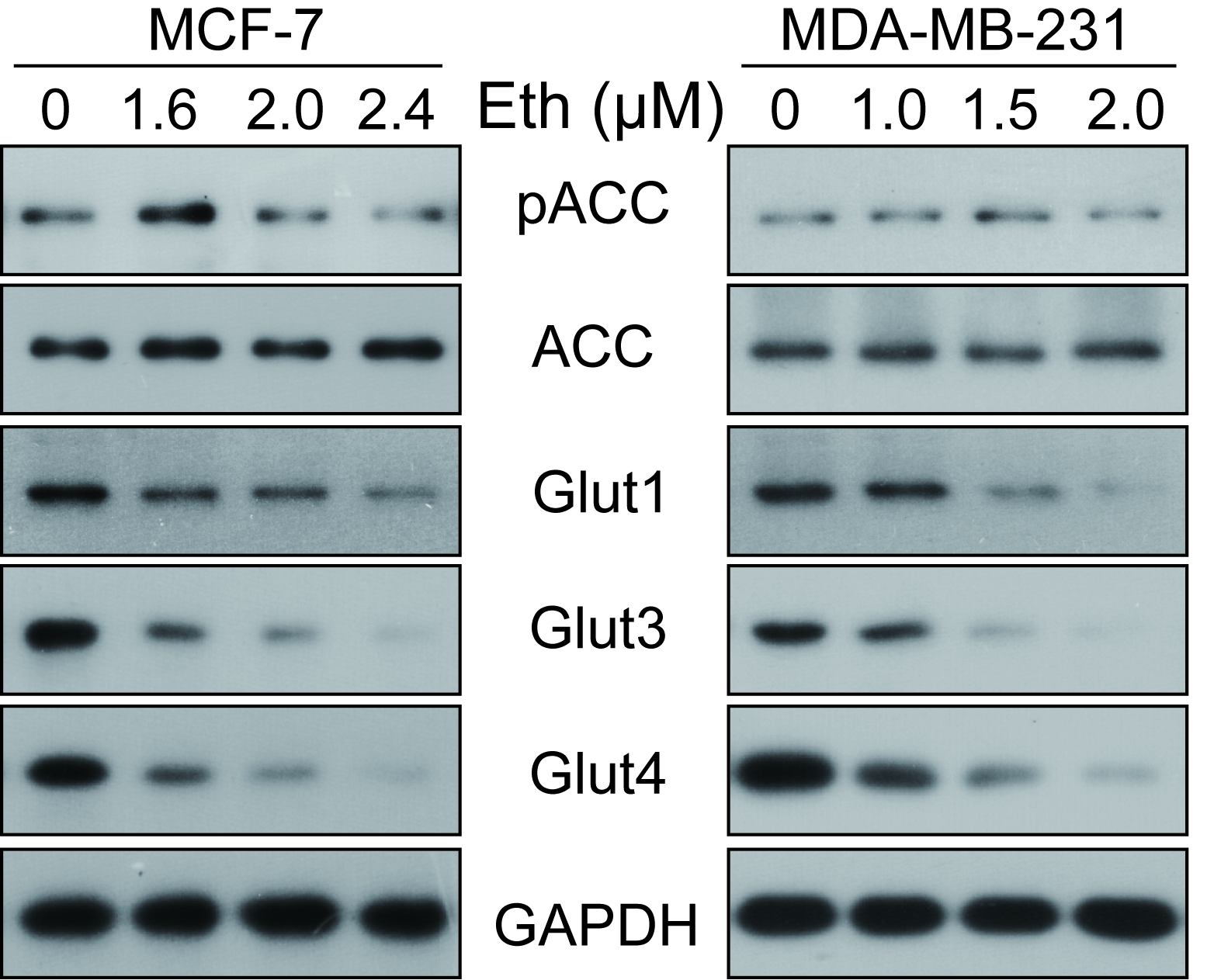

Supplement: Figure S2 — Eth impairs glucose metabolism in BC cells MCF-7 or MDA-MB-231cells were treated with increasing concentrations of Eth for 24 h. Western blot wasperformed using antibodies indicated. [file Image_2.tif]

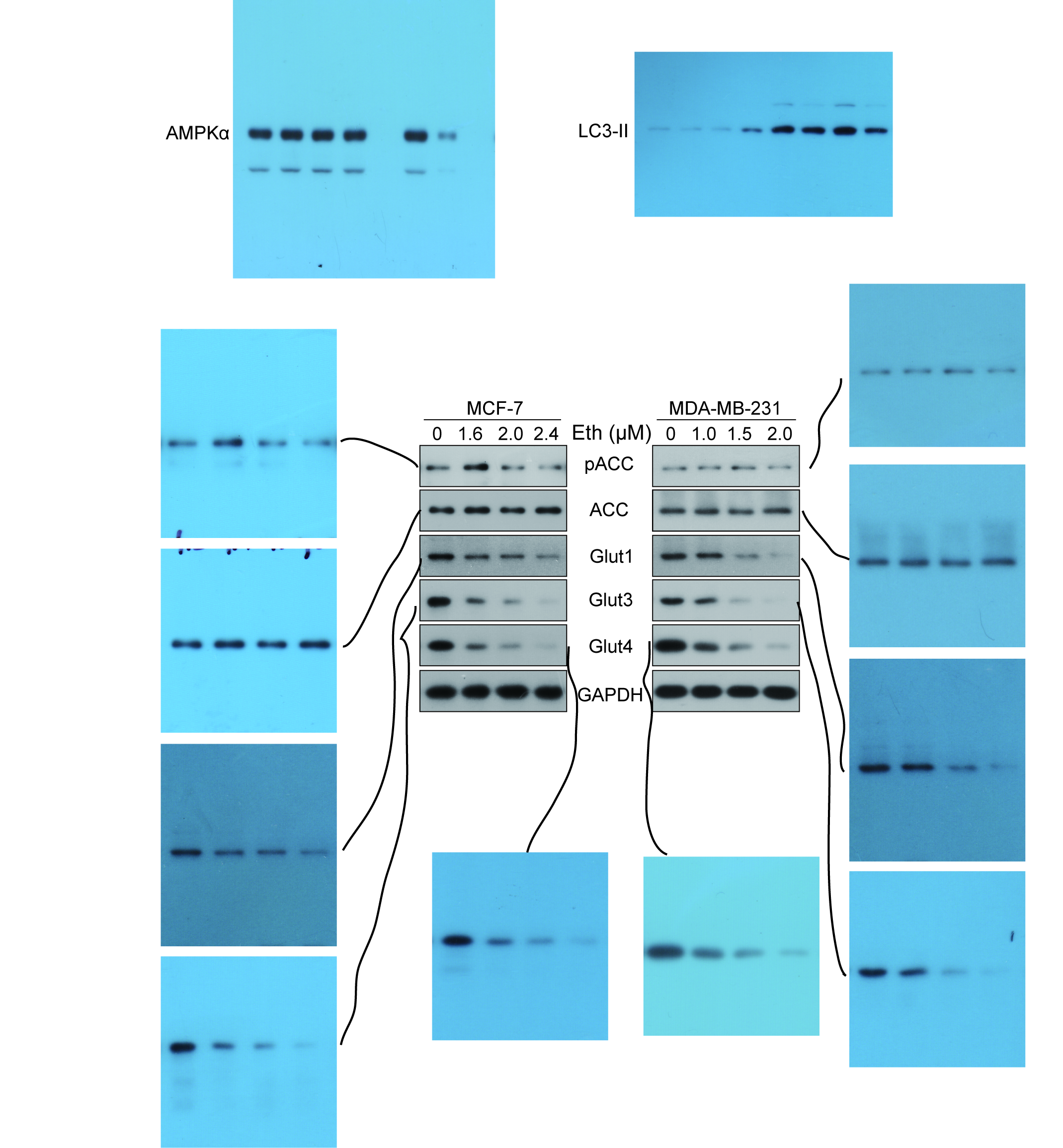

Supplement: Figure S3 — Uncropped immunoblot for some protein. [file Image_3.tif]
